# Variation in crop zinc concentration influences estimates of dietary Zn inadequacy

**DOI:** 10.1371/journal.pone.0234770

**Published:** 2020-07-09

**Authors:** Leah E. M. Bevis, Rachel Hestrin

**Affiliations:** 1 Department of Agricultural, Environmental and Development Economics, The Ohio State University, Columbus, Ohio, United States of America; 2 Soil and Crop Sciences, School of Integrative Plant Science, Cornell University, Ithaca, New York, United States of America; Selcuk University, TURKEY

## Abstract

**Background:**

Zinc (Zn) deficiency is one of the most common micronutrient deficiencies worldwide. Accurate estimates of Zn intake would facilitate the design and implementation of effective nutritional interventions.

**Objective:**

We sought to improve estimates of dietary Zn intake by evaluating staple crop Zn content and dietary Zn consumption by children under the age of 5 in 9 rural districts of Uganda.

**Methods:**

We measured the Zn content of 581 crop samples from household farms and 167 crop samples from nearby markets, and administered food frequency questionnaires to the primary caretakers of 237 children. We estimated Zn consumption using 3 sources of crop Zn content: (i) the HarvestPlus food composition table (FCT) for Uganda, (ii) measurements from household crops, and (iii) measurements from market crops.

**Results:**

The Zn content of staple crops varied widely, resulting in significantly different estimates of dietary Zn intake. 41% of children appeared to be at risk when estimates were based on market-sampled crops, 23% appeared at risk when estimates were based on the HarvestPlus FCT, and 16% appeared at risk when estimates were based on samples from household farms.

**Conclusion:**

The use of FCTs to calculate Zn intake overestimated the risk of dietary inadequacy for children who primarily consumed staple crops that were produced on household farms, but underestimated the risk for children who primarily consumed staple crops that were purchased at market. More information on the Zn content of staple crops in developing countries could lead to more accurate estimates of dietary intake and associated deficiencies.

## 1 Introduction

Zinc (Zn) deficiency is widespread and has severe global health implications. Zn deficiency is associated with poor birth outcomes, reduced growth and cognitive development of infants and children, increased diarrhea and acute lower respiratory infections, and compromised immune function [1, 2, 3]. At least 17% of the global population is at risk of Zn deficiency, with the highest burden in Sub-Saharan Africa [4]. Children are particularly vulnerable to Zn deficiency: 116,000 child deaths in 2011 were attributed to Zn deficiency [5] and Zn supplementation is one of the most impactful nutrient interventions for reducing child mortality [6].

To design and implement targeted policies to improve human Zn nutrition, it is necessary to accurately estimate Zn deficiency rates within a given population. Representative samples of individual blood plasma or serum Zn concentrations can be used to estimate the risk of Zn deficiency in populations [7, 8]. However, the sampling and measurement of blood Zn concentrations at a nationally or regionally representative level is difficult and expensive. Instead, population-level Zn deficiency is often inferred from the prevalence of Zn intake below the estimated average requirement (EAR) [8, 9, 10, 4]. Typically, these estimates are calculated using surveys of a population’s dietary habits or national food balance sheets, and the “average” Zn content of these foods as reported in food composition tables (FCTs) [11].

However, several studies have shown that estimates of population-level Zn deficiency rates based on nationally-representative measurements of blood Zn concentration do not correspond with population-level estimates of inadequate dietary Zn intake. In a study of 20 middle- or low-income countries for which nationally-representative data were available, Zn deficiency rates based on plasma or serum Zn concentrations were poorly correlated with estimates of dietary Zn inadequacy rates, which generally underestimated the number of women and children who were Zn deficient [12]. Similar incongruity has been found in other studies. For instance, of women surveyed throughout Cameroon, those in the north reported the highest Zn intake via a 24-hour dietary recall assessment but had the lowest plasma Zn concentrations in the country [13]. In China, estimated dietary Zn inadequacy in children rose by a factor of 5 between 2002 and 2012, while estimated deficiency rates based on serum samples dropped by a factor of 4 [14]. Similar discrepancies have been observed in Serbia [15], Australia [16], Bangladesh [17], India [18], and Benin [19]. These discrepancies are partially due to variation in Zn bioavailability, which is dependent on phytate intake, gastrointestinal health, and other complex physiological factors that remain poorly understood [7, 20, 21]. Discrepancies may also be due to uncertainty regarding an individual’s physiological requirement for absorbed Zn [22].

Here, we postulate that discrepancies between population-level estimates of Zn deficiency based on blood Zn concentrations versus dietary Zn intake may also be explained by a third factor: a large variation in food Zn concentrations, which are not currently captured by the FCTs used to estimate dietary Zn intake. Although it is well known that environmental conditions and agricultural practices drive heterogeneity in crop Zn concentrations [23, 24, 25, 26], the values reported in most FCTs are based on Zn concentrations measured in food samples collected in the United States or western Europe, rather than samples representative of the food supply available in the particular country of interest [27, 28, 29]. For instance, almost all of the nutrient concentrations reported in the HarvestPlus FCT for Uganda are based on the values reported in the United States Department of Agriculture’s FCT. To our knowledge, Ethiopia is the only African country for which there is an FCT based on nutrient contents measured in crop samples collected from within the country, but Zn concentrations are not reported in this FCT [30].

Thus, there are three major factors that limit accurate predictions of dietary Zn adequacy: 1) unknown variation in Zn consumption due to unobserved heterogeneity in crop Zn concentrations, 2) unknown rates of Zn absorption due to variation in Zn bioavailability, and 3) uncertainties in the physiological requirements for absorbed Zn. No published work to date has investigated the first factor: the effect of crop Zn heterogeneity on dietary Zn intake. Although some scientists and practitioners recognize that the food nutrient concentrations reported in FCTs may be inaccurate [19, 31], most authors using FCTs to infer nutrient deficiencies do not acknowledge or address this limitation directly. The degree of error introduced by these FCTs has not been previously quantified and the implications of the potential error are unknown.

In this paper, we examine staple crop Zn heterogeneity in rural Uganda and explore how this heterogeneity affects estimates of Zn consumed by individual children and subsequent estimates of dietary Zn inadequacy at the population level. To this end, we employ a unique dataset that combines household survey data, Zn concentration measured in staple crops collected from household farms in 9 rural districts of Uganda, and food intake data for children living in households engaged in subsistence agriculture. Using food frequency questionnaire data to assess patterns of consumption for children under the age of 5, we estimate dietary Zn intake and Zn inadequacy rates based on three different sources of staple crop Zn concentrations: 1) the HarvestPlus FCT created for Uganda, 2) the Zn values measured in 581 food samples that were grown by households engaged in subsistence agriculture in 9 rural districts of Uganda, and 3) the Zn values measured in 167 food samples purchased from 32 local markets throughout the same 9 districts. Finally, we compare the resulting estimates to explore the degree to which Zn intake is accurately estimated by existing FCTs.

## 2 Materials and methods

### Ethics statement

The University of North Carolina at Chapel Hill Institutional Review Board approved the interview protocol used in the study (approval no. 12–1189), and documented that informed, oral consent was ethically obtained and that the anonymity of participants’ responses was maintained. Brown University, Purdue University, the International Good Policy Research Institute and Cornell University were approved as collaborating institutions. Participants provided oral consent, which was deemed sufficient due to the minimal risk posed by the study.

### Data

**Fig 1:**
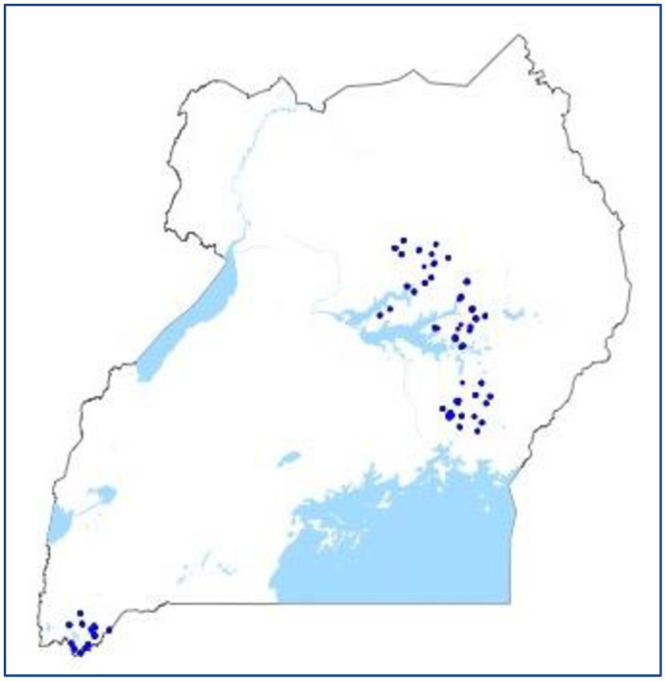
Crop sample locations.

Household survey data were collected from 9 districts of the western, northern, eastern and lakes regions of Uganda during the summer of 2013 [32]. Households included in the survey were engaged in subsistence agriculture. Crop samples were collected from household farms at the time of survey. Six staple crops were sampled from each household, if present: maize, sorghum, sweet potato, cassava, beans and groundnuts. A total of 581 crop samples were collected from 282 households in the weeks during and after harvest. The locations of these 282 households is shown in Fig 1. Only crops produced on a household’s land were included in household-level samples; stored crops purchased from market were never sampled from households. For grains and legumes, surveyors subsampled crops from ten locations within each plot. Because cassava and sweet potato are less easily divisible, surveyors subsampled these crops from five or six plants within each plot. The total mass of each composite crop sample collected from a plot was equal to one kg or more. This sampling scheme was chosen to obtain a representative sample of the crop Zn content within each plot [33, 34, 10, 35].

Food samples were also collected from the rural markets nearest these households. Maize flour, cassava flour, millet flour, cowpeas, white rice, and matooke (a cooking banana used as a staple) were collected from markets. In total, 167 samples from 32 markets were collected. Together, the crops collected from both households and markets include all primary staple crops consumed in Uganda.

Prior to nutrient analysis, all samples aside from the flour samples were brushed and washed with distilled water to remove soil and dust particles, air-dried, ground to pass through a 2.0 mm sieve using a stainless steel mill, and homogenized. Subsamples of 0.5 g were digested in 5.0 mL of nitric acid and 2.0 mL of perchloric acid. The elemental composition of digested subsamples was measured using an axially viewed Spectro Arcos ICP-AES (Spectro Arcos, Kleve, Germany). Blanks and standard reference materials were run throughout in order to ensure consistency and quality of the ICP-AES analysis. For Zn, three positions were monitored, located at 202.613, 206.200, and 213.856 nm. Visual inspection of the signal at each position ensured that measurements were not impeded by interference between elements. Our subsequent analysis is based on the Zn concentrations measured at 206.200 nm because this position is less prone to fluctuations in plasma conditions. Additionally, Yttrium was used as an internal standard, to correct for instrument drift and matrix interferences. The limit of detection for Zn at 206.200 nm was 0.00308 parts per million (ppm). This is far below the lowest Zn concentration measured among our samples. The Zn concentration measured in 4 samples (2 sweet potato and 2 groundnut samples) was higher than commonly reported for similar crops. Although all samples were washed and brushed prior to analysis, a few samples may have contained soil or dust particles, which can have high Zn concentrations compared to plant-based foods. However, because our Zn intake analysis is based on median Zn concentrations (or other percentiles), the relatively few samples containing very high Zn do not influence our statistical analysis or conclusions.

**Fig 2:**
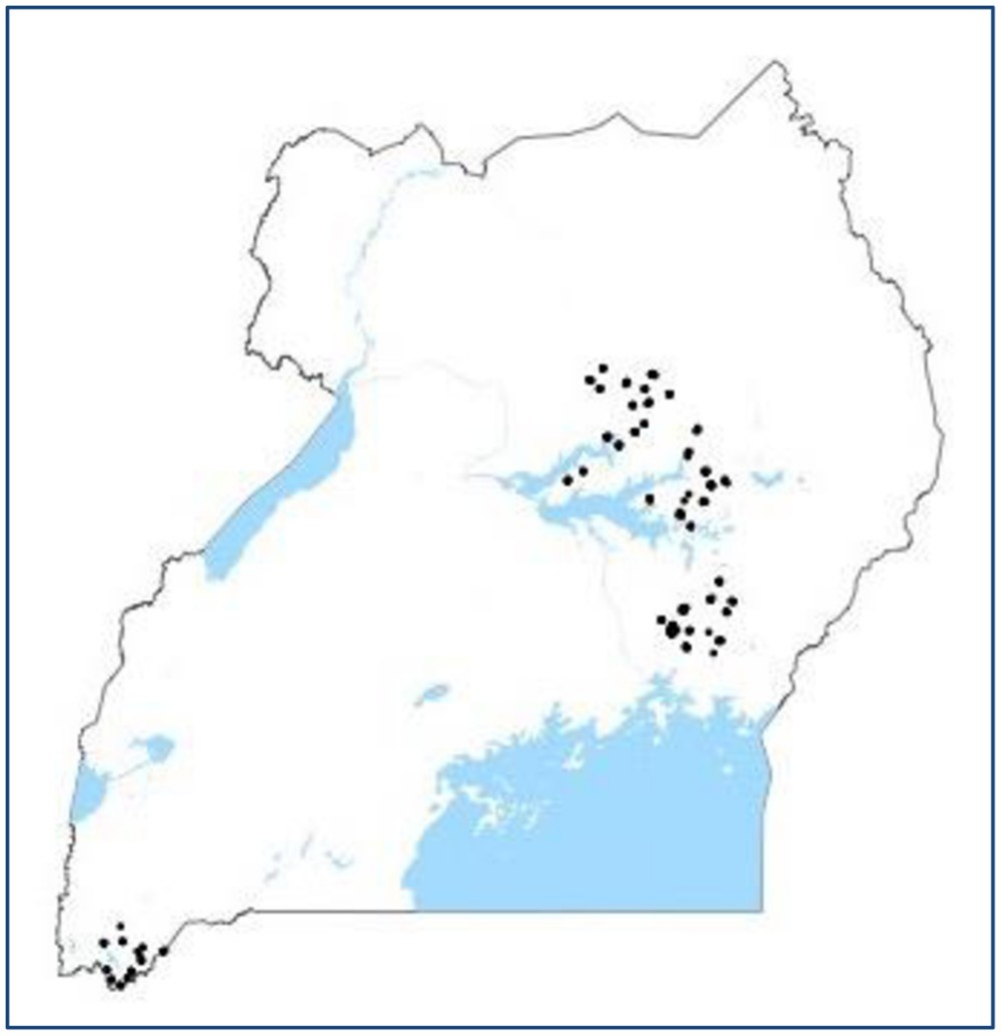
FFQ locations.

A food frequency questionnaire assessing dietary Zn intake was conducted for one child per household, in 231 households. The locations of these households is shown in Fig 2. The similarity between Figs 1 and 2 highlights the geographical overlap of crop samples and child food intake data. Children selected for the questionnaire were between 6 months and 5 years of age, present at the time of the survey, and not exclusively breastfed. If multiple children in a household fit these criteria, surveyors chose the oldest biological child of the household head. The food frequency questionnaire was designed and validated to assess Zn intake using 24-hour food recall data gathered in central and eastern Uganda by HarvestPlus for children under 5 [36]. The validation procedure followed a HarvestPlus technical document [37]. Fifty-two commonly consumed plant- and animal-based foods were included in the questionnaire, in order to capture 98% of total Zn intake in the 24-hour recall data. Because the 2013 food frequency questionnaire included districts in western Uganda as well as in central and eastern Uganda, the food list was expanded to contain a few dishes consumed in western Uganda. Portion sizes (small, medium and large) were chosen as the 25th, 50th, and 75th quantile of portion size weight in the food recall data. Because the food frequency questionnaire was designed and validated only to assess Zn intake, the data captured by the questionnaire may not be sufficient to assess the total intake of any other macro- or micronutrient.

The questionnaire, shown in S1 Table, was administered to the primary caregivers of the 231 selected children. These caregivers were asked to report how many times their child had consumed each food in the preceding week and the average portion size of each food consumed. To assist caregivers in their estimates of portion size, photographs of small, medium, and large portions were provided (see examples in S1 and S2 Figs). The plate depicted in the photograph was brought to each home for scale. In 99 cases, a second food frequency questionnaire was administered within 1–4 weeks of the first, allowing us to assess weekly variation in an individual child’s diet.

### Statistical analysis

To examine heterogeneity in crop Zn concentration, we used univariate kernel density estimation to estimate the distribution of Zn concentration for each of the staple crops collected. We performed this analysis in Stata using the kdensity command, an Epanechnikov kernel function, and the optimal bandwidth choice to minimize mean squared error. We compared the distributions of the Zn concentrations measured in cereals, legumes, and tubers sampled from household farms, and tested their equivalence using a Kolmogorov-Smirnov test. We also compared the distributions of the Zn concentrations measured in staples sampled from markets. In three cases where comparable (though not identical) staples were sampled at the household and market level, we compared the Zn concentration distributions of household-sampled staples to market-sampled staples, and tested their equivalence: maize grain vs. maize flour, sorghum grain vs. millet flour, and cassava tubers vs. cassava flour. Alongside these distributional assessments, we also examined summary statistics (minimum, maximum, median, mean, and standard deviation) for the Zn concentration of each crop. For each crop, we compared the mean Zn concentrations to the FCT values using a single sample t-test, and the median Zn concentrations to the FCT values using a single sample Wilcoxon test.

We estimated dietary Zn intake (mg day−1) for each child using 3 sources of values for staple crop Zn concentration: (1) those reported in the HarvestPlus FCT, (2) the median concentrations measured in crops sampled at local markets, and (3) the median concentrations measured in crops sampled from household farms. The second estimate, based on Zn values measured in crops from local markets, represents the risk of dietary Zn inadequacy for children who primarily consume staples purchased at market. The third estimate, based on Zn values measured in crops from household farms, represents the risk of dietary Zn inadequacy for children who primarily consume staples produced by households engaged in subsistence agriculture. Since the Zn concentrations of crops sampled from households were quite heterogeneous, we also calculated two additional estimates of Zn intake to represent variation in the potential range of dietary Zn intake from crops produced on household farms. These two additional estimates were calculated using the 25th and 75th percentile of Zn concentrations measured in samples collected from households. It is worth noting that if particularly high measurements of crop Zn concentration were due to soil contamination, these outliers would not appreciably affect our Zn intake estimates, since quartile values are not strongly influenced by a few high outliers. S2 Table outlines the sources of staple Zn concentration used to calculate each of the Zn intake estimates. When estimating Zn intake based on crops purchased at market, we replaced the FCT Zn concentrations of maize grain and flour, sorghum/millet grain and flour, cassava tubers and flour, rice, matooke, and cowpeas with the median Zn concentrations measured in maize flour, millet flour, cassava flour, rice, matooke, and cowpea samples collected from local markets. When estimating Zn intake based on crops produced at households, we replaced the FCT Zn concentrations of maize grain and flour, sorghum/millet grain and flour, cassava tubers and flour, beans, groundnuts, and sweet potatoes with the median Zn concentrations measured in maize grain, sorghum grain, cassava tubers, beans, groundnuts, and sweet potatoes collected from households. For both estimates, we used the Zn concentrations reported in the HarvestPlus FCT to calculate Zn intake from all foods that were not sampled in the relevant location. The HarvestPlus FCT applies USDA retention codes to account for nutrient loss due to cooking processes. We applied the same retention codes to account for Zn loss from cooked staples in the Zn intake estimates based on market-purchased and household-produced foods.

A notable limitation of our data is that the types of staples sampled from households were not identical to those sampled from markets. In particular, whole maize and sorghum grain were sampled from households, while maize and millet flour were sampled from markets. To address these discrepancies, first we combined the quantity of millet and sorghum consumed and assigned one Zn concentration to the total quantity consumed of both crops (S2 Table). For household-based estimates, this value was based on the Zn concentration measured in sorghum grain sampled from households. For market-based estimates, this value was based on the Zn concentration measured in millet flour sampled from markets. Although separate measurements of each staple food sourced from either the market or household would have been preferable, previous documentation of millet and sorghum Zn concentrations suggest that they are similar to each other. The HarvestPlus FCT lists the same Zn concentration for both sorghum and millet. Zinc concentrations measured in finger millet—the primary form of millet grown in Uganda—are similar to those measured in sorghum [38, 39, 40]. Pooling the two staples for our estimates provides a market-based counterfactual for sorghum consumption and a household-based counterfactual for millet consumption.

Second, we reduced the Zn concentration of household-sampled whole maize grain by 50% when it was consumed as maize flour (S2 Table). Most families in rural Uganda process their maize grain at local mills prior to consuming it as flour. This process often results in nutrient loss from the original grain. A wide range of Zn loss can occur when whole maize grain is processed into flour: a study in Benin reported an 11% decrease in Zn when maize was ground into flours at local mills [41], Zn content decreased by 18% when maize was ground into the course flour product masa [42], while up to 57% of Zn was lost when maize flour was refined in Malawi [43]. FCTs also report variable differentials between the Zn concentration of maize grain and maize flour [44, 27]. We chose to reduce the Zn concentration of maize that was produced on household farms but consumed as flour by 50% because this provides a statistically conservative estimate of dietary Zn intake. We did not reduce the Zn concentration of household-grown millet and sorghum when they were consumed as flour because studies have shown that milling does not result in appreciable Zn loss from these crops [45, 46, 47, 48]. We did not adjust the Zn concentration measured in household-grown cassava, because prior to Zn measurement, household-grown cassava was peeled, sliced, dried and ground in the same way that market-sampled cassava flour would have been, which likely resulted in a similar degree of nutrient loss for both.

Thirty-nine of 231 children, aged 6.7–53.2 months, were still breastfeeding at the time of interview. Because breastfed children consumed a portion of dietary Zn through breastmilk, we adjusted Zn intake estimates for breastfeeding status according to the observed average, age-specific difference in Zn intake between breastfeeding and non-breastfeeding children. This data-driven technique uses non-breastfeeding children as “controls” for breastfeeding children, allowing Zn intake to be adjusted for breastfeeding status without the need to account for breastmilk Zn concentrations or the quantity of breastmilk consumed by any given child. To implement this adjustment, we regressed Zn intake on age in months, breastfeeding status (a binary variable), and an interaction between the two, and then adjusted intake up according to the age-specific effect of breastfeeding. Although the average age of children who were breastfeeding was lower than the age of those who were not, there was sufficient overlap to calculate the age-specific effect of breastfeeding. Because a non-linear effect of age did not contribute to a higher R2, we retained the linear model.

The food frequency questionnaire was administered a second time to the caregivers of 99 children a few weeks after the first questionnaire. Intra-subject variation was negligible, accounting for only 5% of total variation. This suggests that the food frequency questionnaire successfully captured “usual” Zn intake for children under the age of 5 in the households sampled. However, to gauge the effect of intra-subject variability, we adjusted intake following the simplified Nusser procedure proposed by Hoffmann et al. [49]. The procedure was performed in Stata. The distributions of the usual intake estimates created through this procedure were slightly less variable than the original estimates of Zn intake. For example, the adjusted intake based on Zn values reported in the HarvestPlus FCT had a standard deviation of 17.39, while unadjusted intake had a standard deviation of 18.96. However, the change in distribution was so slight that the Nusser adjustment did not result in a difference in the predicted rate of dietary Zn inadequacy for estimates based on the HarvestPlus FCT or based on crops sampled from market, and resulted in only a 1% difference for the estimate based on crops sampled from households. We therefore used the original Zn intake estimate for our main analysis, averaging the two Zn intake estimates for children from whom the food frequency questionnaire data was collected twice.

We estimated the prevalence of inadequate dietary Zn intake using the cut-point method and the age-specific EAR recommended by International Zinc Nutrition Consultative Group (IZiNCG) for unrefined cereal-based diets [7]. High levels of phytate intake associated with unrefined cereal-based diets are thought to reduce Zn absorption substantially [7]. Since we did not measure the quantity of phytates consumed by each child, we were unable to adjust for variation in Zn bioavailability due to phytates. However, because the diets of all children in this study were cereal-based, the EARs formulated for cereal-based diets were deemed appropriate. We bootstrapped the standard errors around prevalence of inadequate dietary Zn intake predicted from each of the three sources of staple crop Zn concentrations: the values reported in the HarvestPlus FCT for Uganda, the staple crops sampled from local markets, and the staple crops sampled from households engaged in subsistence farming.

## 3 Results

### Crop Zn heterogeneity

**Fig 3:**
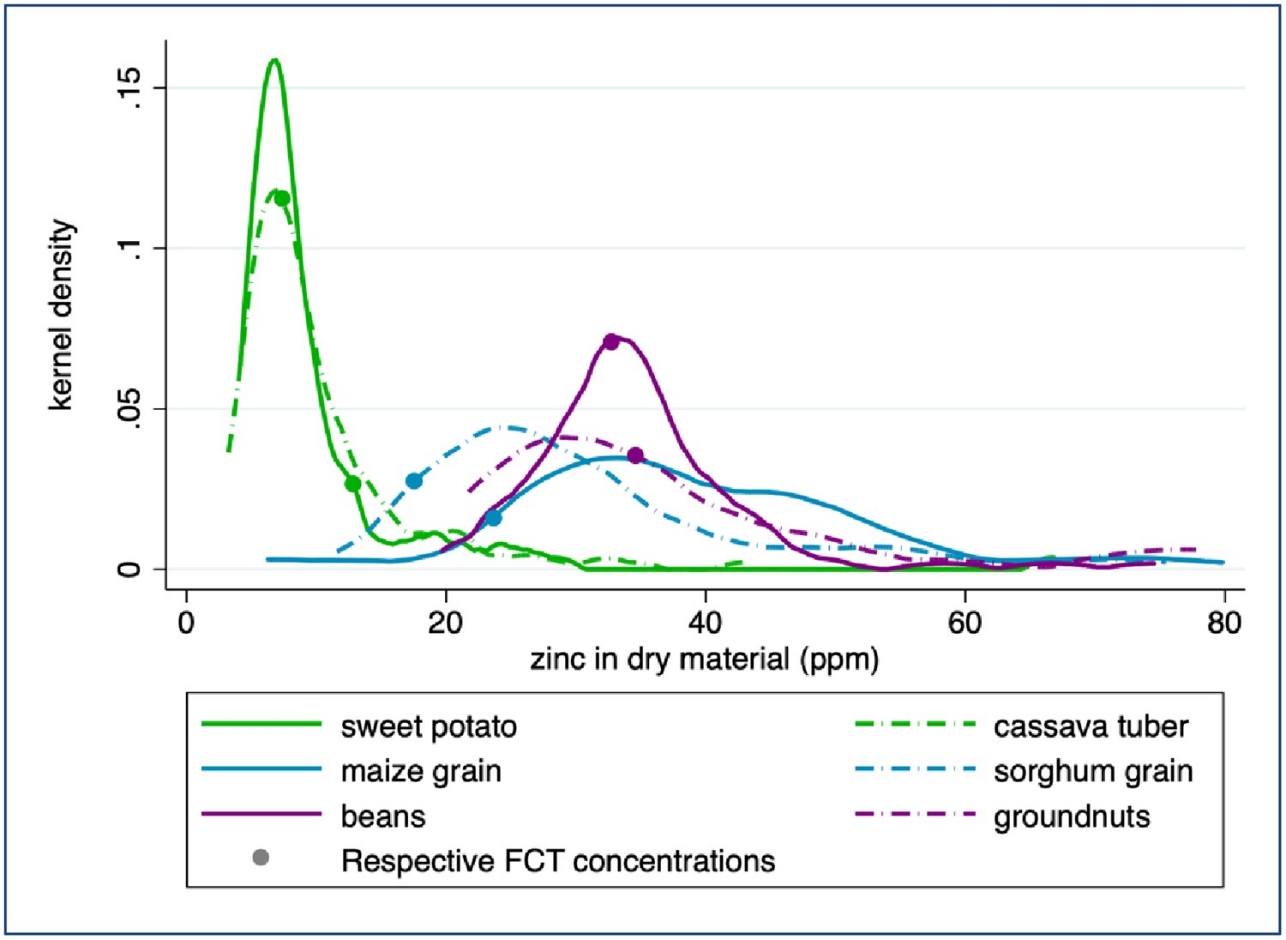
Zinc concentration in household farm samples. Values exceeding 80 ppm are not displayed. FCT sources are listed under Table 1.

The Zn concentrations of staple crops grown and consumed within a household in Uganda were highly heterogeneous within each crop type. The maximum Zn concentrations measured in sorghum and maize were 16 and 20 times higher than the minimum concentrations measured, respectively (Fig 3 and Table 1). The log-normal distribution of crop Zn concentration suggests that median Zn concentration values are more representative than the average Zn concentration values that are usually reported in FCTs and used to estimate dietary Zn intake. Although the Zn concentration of legumes is thought to be higher than that of cereals [27, 11]), the distributions measured in our samples were similar: median Zn concentrations of sorghum, beans and groundnuts were within half a standard deviation of the median Zn concentration of maize (Table 1). Median Zn concentration in legumes (33.75 ppm) was only slightly higher than median Zn concentration in cereals (31.37 ppm); the difference was only marginally significant (Wilcoxon test p = 0.056). Conversely, both the median and the distribution of tuber Zn concentration were significantly different from those of other crops (p < 0.001 using a Wilcoxon and Kolmogorov-Smirnov test, respectively).

**Table 1:** Zn concentration measured in staple crops collected from household farms (ppm dry mass; 10 ppm = 1 mg 100 g^-1^).

	Household Sample Value						
						FCT	% <
	Min	Median	Mean	Max	Sdev	Value	FCT
Maize grain	6.3	38.3***	42.3***	127.5	19.9	23.8	7.1
Sorghum grain	11.6	28.2***	34.1**	184.6	23.9	17.6	6.5
Sweet potato	4.2	7.7***	10.7	90.2	11.7	12.9	85.1
Cassava	3.2	7.9	10.0	43.4	6.5	7.4	45.4
Beans	19.7	33.7	34.5	74.5	8.3	32.8	42.4
Groundnuts	21.7	34.0	61.4	427.6	80.8	34.7	50.0

Stars denote significant differences compared to FCT values using a t-test for mean values and a Wilcoxon test for median values (*** p < 0.01, ** p < 0.05, * p < 0.1). Sources for FCT values: HarvestPlus 1001/1004 from USDA-21 20014 (maize grain), HarvestPlus 1105 from WFDA 1010 (sorghum grain, Senegal), HarvestPlus 3003 from USDA-21 11507 (sweet potato), HarvestPlus 2001 from USDA-21 11134 (cassava), HarvestPlus 6121 from USDA-21 16027 (beans), and HarvestPlus 8015 from USDA-21 16087 (groundnuts).

According to a Wilcoxon test, the HarvestPlus FCT for Uganda does not represent the median crop Zn concentrations of 4 of the 6 staple crops sampled from household farms in our study. The median Zn concentration of sweet potatoes produced on household farms was 40% lower than reported by the HarvestPlus FCT. The median Zn concentrations of maize and sorghum produced on household farms were both 60% higher than the values reported in the HarvestPlus FCT. The Zn concentration of cassava reported in the FCT is only 6% lower than the concentration measured in household samples, but this difference is significant (p = 0.003). For the remaining 2 staple crops collected from households—groundnuts and beans—the Zn concentrations measured in our samples correspond with the values reported in the HarvestPlus FCT.

**Fig 4:**
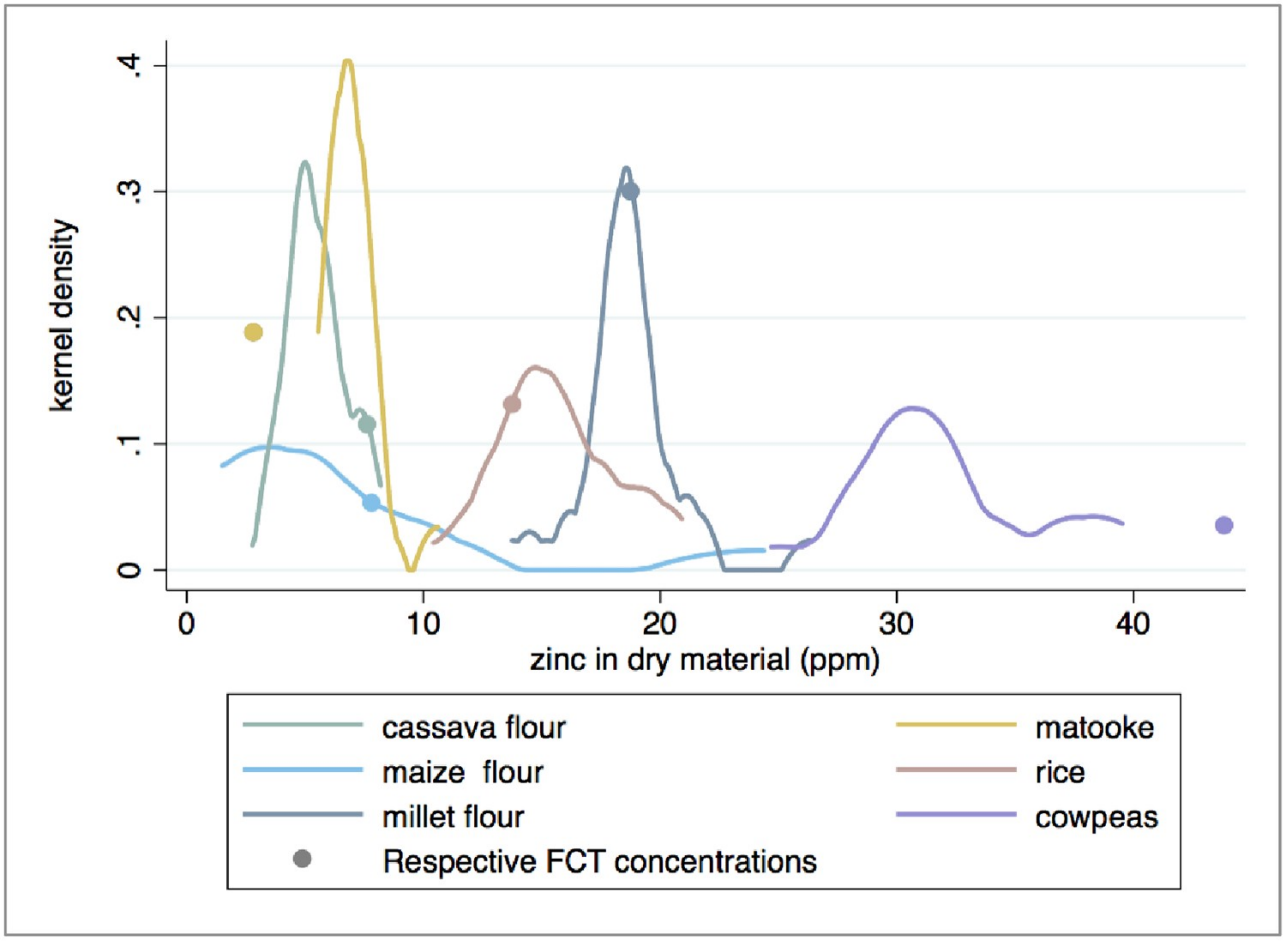
Zinc concentration in market samples. FCT sources are listed under Table 2.

The Zn concentrations of market-sampled crops were less heterogeneous than those collected from household farms, but again the FCT does not represent the median Zn concentrations of 4 of the 6 crops. The mean Zn concentration measured in our samples (6.6 ppm) is statistically indistinguishable from the mean concentration of 5.6 ppm reported by Tidemann-Andersen et al. [50] for maize flour samples purchased from Ugandan markets (p = 0.411), once their figures are adjusted to provide ppm dry matter. (Tidemann-Andersen et al. [50] report sample values as 4.2, 3.6, and 7.0 ppm wet matter. According to the reported moisture content of maize flour, this is equivalent to 4.77, 4.09, and 7.95 ppm dry matter, resulting in a mean value of 5.6 ppm and a median value of 4.8 ppm.) However, due to the skewed distribution of the Zn concentrations (Fig 4), the median value provides a better representation of the “typical” maize flour found at market than the mean, and that sample median value is almost half of the sample average value (Table 2). The median Zn concentration of matooke samples collected from markets was more than twofold higher than the value reported in the HarvestPlus FCT (Table 2). However, the median Zn concentrations of maize flour, cassava flour, and cowpea samples collected from markets were approximately 50%, 70%, and 66% lower than the values reported in the FCT, respectively (Table 2 and Fig 4). The median Zn concentrations of millet flour and rice measured in our samples correspond with the values reported in the HarvestPlus FCT.

**Table 2:** Zn Concentration measured in staple crops collected from markets (ppm dry mass; 10 ppm = 1 mg 100 g^-1^).

	Household Sample Value						
						FCT	% <
	Min	Median	Mean	Max	Sdev	Value	FCT
Maize flour	1.5	3.8	6.6	24.4	6.5	7.9	70.0
Millet flour	13.7	18.6	18.7	26.4	2.3	18.8	58.6
Cassava flour	2.8	5.3	5.5	8.2	1.3	7.7	93.5
Cowpeas	24.7	30.5	32.0	39.5	4.0	43.9	100.0
White rice	10.4	15.5	15.6	20.9	2.6	13.8	19.4
Matooke	5.6	6.8	7.0	10.6	1.1	2.9	0.0

Stars denote significant differences compared to FCT values using a t-test for mean values and a Wilcoxon test for median values (*** p < 0.01, ** p < 0.05, * p < 0.1). Source for FCT values: HarvestPlus 1041 from USDA-21 20522 (maize flour), HarvestPlus 1104 from USDA-21 20031 (millet flour), HarvestPlus 2020 from WFDA 10120 (cassava flour, Indonesia), HarvestPlus 6211 from USDA-21 11191 (cowpeas), HarvestPlus 1201 from USDA-21 20450 (white rice), and HarvestPlus 5001 from USDA-21 9277 (matooke).

**Fig 5:**
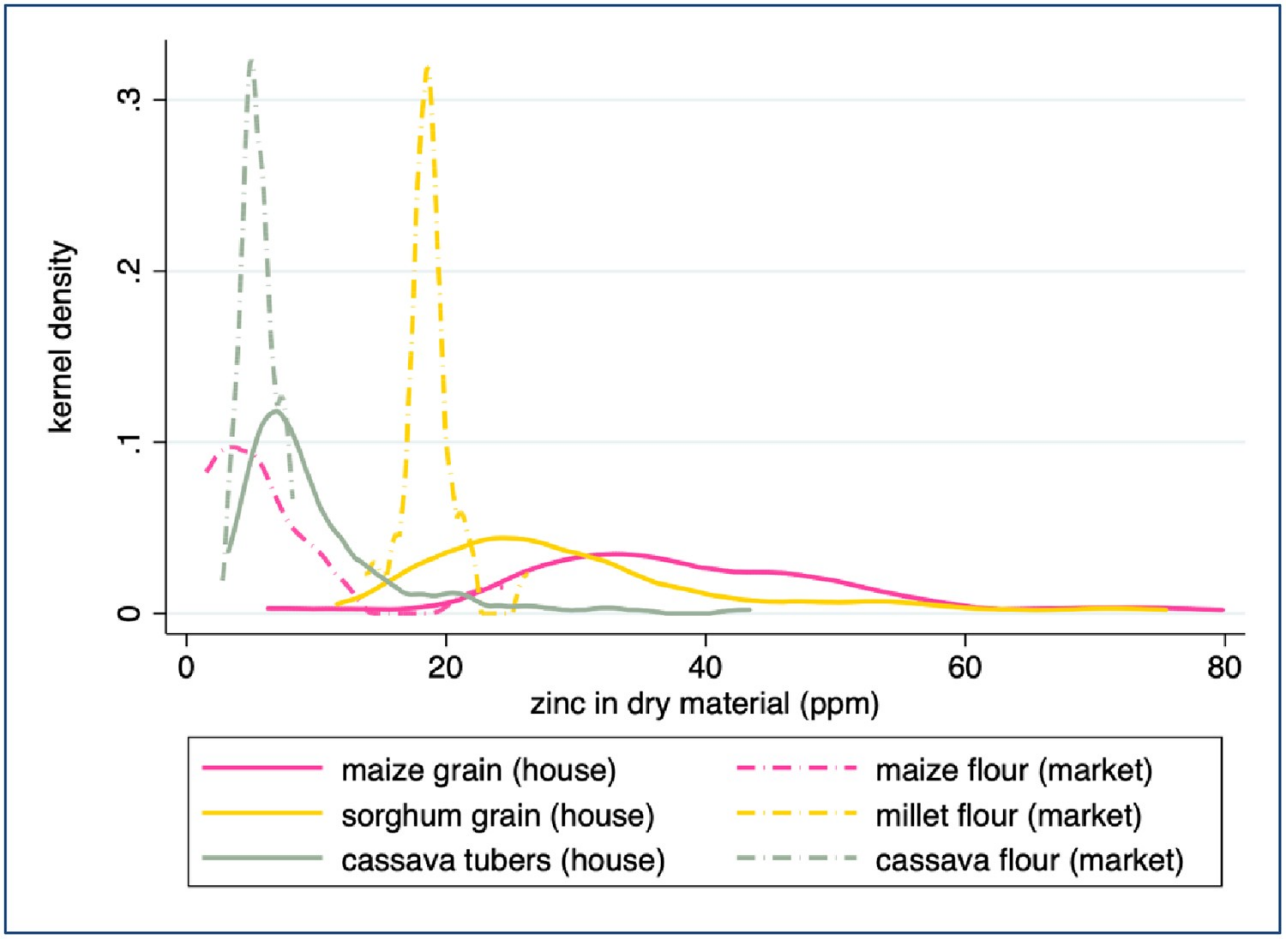
Zinc concentration in comparable farm-market samples. Values exceeding 80 ppm are not displayed.

The Zn concentrations measured in flours sampled from market were significantly lower than the Zn concentrations measured in whole foods sampled from household farms (Fig 5, p < 0.001 for each pair based on either a two sample t-test of means or a two sample Wilcoxon test of median equivalence). Most notably, the Zn concentration of maize flour sampled from market was only 10% of the median Zn concentration measured in whole maize grain collected from household farms (3.8 and 38.3 ppm Zn, respectively). Between 11–60% of this difference may be due to processing, which removes part or all of the grain germ and pericarp [43, 41, 28]. The remaining difference between Zn concentration in maize grain and in flour samples may be due to an underlying difference in nutrient concentrations of crops grown on household farms and sold at local markets. The Zn concentrations of cassava flour collected from markets was 30% lower than that of cassava tubers collected from household farms. Since the cassava tubers sampled from household farms were peeled and dried in the laboratory, as they would have been prior to being ground into flour for market, processing loss should not be responsible for the observed difference in Zn content. Similarly, the 33% decrease in median Zn concentration of millet flour compared to whole sorghum grain sampled on farms may be due to nutrients lost during milling, but generally such loss is not observed for sorghum and millet [45, 46, 47, 48]. In all three cases, consumption of the whole crop or of whole-grain flour rather than refined flour would result in higher dietary Zn intake.

### Implications for child Zn intake estimates

The results of the food frequency questionnaire suggest that the majority of both the caloric intake and Zn intake by children participating in this study are derived from staple crops (Table 3). Estimates based on our food frequency questionnaire and HarvestPlus FCT Zn concentrations suggest that cereals alone contributed 38% of total caloric intake and 33% of total Zn intake for the median child surveyed in our study. Legumes, nuts, and seeds provided 16% of the calories and 28% of the Zn consumed. Tubers, generally considered minor contributors to mineral intake, contributed 19% of the calories and 11% of the Zn consumed. The median child in our study consumed 85% of her caloric intake and 84% of her Zn intake through a combination of cereals, legumes, nuts, seeds, and tubers. Animal-based foods contributed only 4% of estimated calorie intake and 9% of Zn intake for the median child, most of which was due to milk and fish consumption. Although exact estimates of calorie and Zn intake vary depending on the method and data source used to calculate consumption rates, any approach would likely indicate that staple crops are the primary source of calories and Zn in children’s diets in rural Uganda.

**Table 3:** Source of calorie and Zn intake according to harvestplus FCT.

	% Kcal intake Median Child	% Zn intake Median Child
Cereal intake (HarvestPlus FCT)	37.9	33.4
Nuts, legumes, and seeds intake (HarvestPlus FCT)	15.9	28.0
Tuber intake (HarvestPlus FCT)	18.7	10.9
Plant-based food intake (HarvestPlus FCT)	84.7	83.6
Animal-sourced food intake (HarvestPlus FCT)	4.0	8.7
Total kcal intake for median child (HarvestPlus FCT)	6,261	

The values reported here are based on a Food Frequency Questionnaire, which was designed and validated only to assess Zn intake. Therefore, total calorie intake is not represented and the relative contribution of each food group to total calorie intake may vary from the values reported here. Cereal: Maize, millet, sorghum, rice, or wheat. Nuts, legumes and seeds: beans, groundnut, cowpeas, pigeon peas, or soy peas. Tubers: sweet potato or cassava. Animal-sourced foods: eggs, fish, beef, or milk.

**Fig 6:**
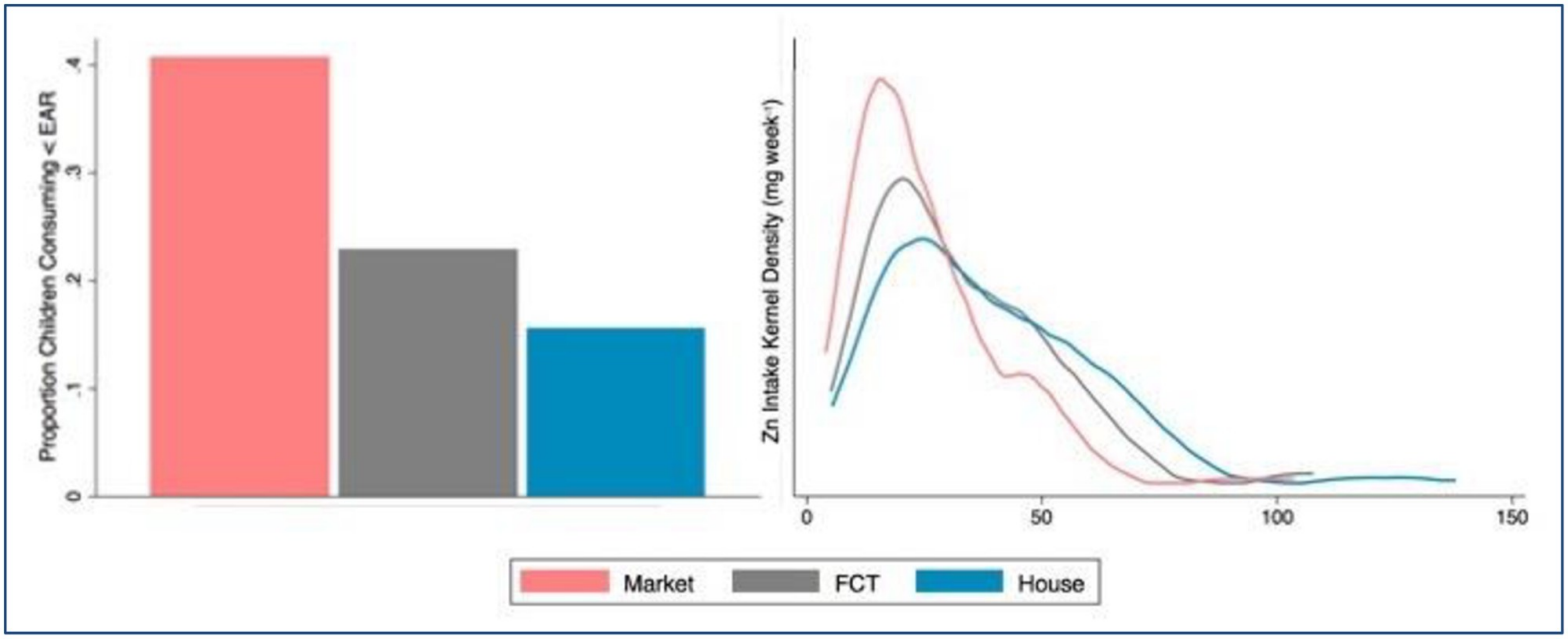
Prevalence of inadequate dietary Zn intake. Left: Proportion of children whose estimated dietary Zn intake is inadequate (mg day^-1^ < EAR). Right: Kernel density distributions for estimated weekly Zn intake. For both graphics, “Market” denotes Zn concentrations measured in samples collected from markets, “FCT” denotes staple Zn concentrations reported in the HarvestPlus food composition table, and “House” denotes Zn concentrations measured in samples collected from households.

Due to a diet that is highly dependent on staple crops for total caloric intake, the population of children participating in this study is vulnerable to heterogeneity in staple crop Zn concentration. Estimates of inadequate Zn intake varied significantly depending on which source of staple Zn content used to calculate dietary Zn intake (Kolmogorov-Smirnov test p < 0.001, Table 4, Fig 6). Estimates of dietary Zn intake based on the HarvestPlus FCT suggested that median Zn intake was 28 mg week−1 and that 23% of children were at risk of inadequate dietary Zn intake. Estimates based on the Zn values measured in samples collected from markets suggested that Zn intake was 21 mg week−1 and that 41% of children were at risk of inadequate dietary Zn intake. Estimates based on the Zn values measured in samples collected from households suggested that Zn intake was 35 mg week−1 and that 16% of children were at risk of inadequate dietary Zn intake. A prevalence of inadequate dietary Zn intake greater than 25% is considered elevated [51]. Thus, our results suggest that children whose primary dietary intake comes from household-produced staples are not at elevated risk for dietary Zn inadequacy, while children whose primary dietary intake comes from market-purchased staples may be at elevated risk for dietary Zn inadequacy.

**Table 4:** Prevalence of inadequate dietary Zn intake.

	Median child mg day^-1^	% children mg day^-1^ < EAR
Zn intake: HarvestPlus FCT Zn concentration	28.1	22.9
Zn intake: Market sample median Zn concentration	20.8	40.7
Zn intake: Household sample median Zn concentration	34.6	16.5

**Fig 7:**
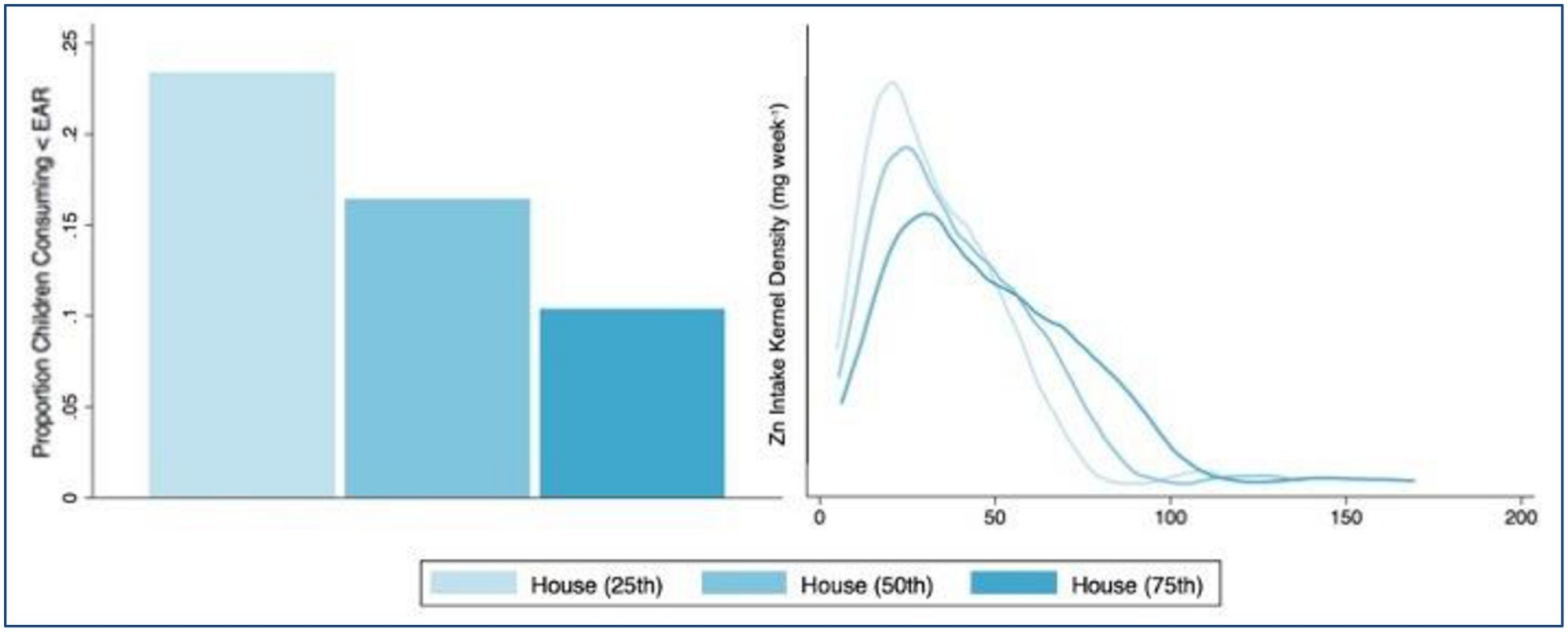
Prevalence of inadequate dietary Zn intake by household Zn concentration quantile. Left: Proportion of children whose estimated dietary Zn intake is inadequate (mg day^-1^ < EAR). Right: Kernel density distributions for estimated weekly Zn intake. In both graphics, the values represent dietary intake estimates based on the 25^th^, 50^th^, and 75^th^ percentiles of Zn concentrations measured in staples collected from household farms.

Even within the population of children who primarily consume staple crops produced at household farms, the risk of inadequate Zn intake varied considerably due to the wide distribution of crop Zn concentrations measured in samples collected from households (Kolmogorov-Smirnov test p < 0.001, Table 5, Fig 7). When Zn intake was calculated according to the 25th percentile of Zn concentration in household-sampled crops, 23% of children appeared to be at risk of inadequate intake. However, only 10% appeared to be at risk of inadequate dietary Zn intake when Zn intake was estimated according to the 75th percentile of household-sampled crops. Intake of animal-based foods was correlated with an increase in calorie and Zn intake via the consumption of both animal- and plant-based foods, for all children participating in the study, thereby decreasing the risk of dietary Zn inadequacy.

**Table 5:** Prevalence of inadequate dietary Zn intake by household concentration quantile.

	Median child mg day^-1^	% children mg day^-1^ < EAR
Zn intake: Household sample 25^th^ percentile	28.5	23.4
Zn intake: Household sample 50^th^ percentile	34.6	16.5
Zn intake: Household sample 75^th^ percentile	43.0	10.4

## 4 Discussion

Our findings suggest that in rural Ugandan households, where meat and eggs are rarely consumed, 84% of dietary Zn intake in children under the age of 5 is consumed from plants-based foods. Over 75% of Zn intake is derived from only 7 staple crops (maize, sorghum, millet, beans, ground nuts, cassava, and sweet potatoes) and over 40% is derived from maize and beans alone. While our data may not be representative of the entire population of children under the age of 5 in Uganda, our data include households that are located in 9 districts of rural Uganda, representing every agro-ecological zone in the country. A similar dependence on plant-based foods is common in much of sub-Saharan Africa and South Asia, often accompanied by low dietary diversity and risk of Zn deficiency [50, 52, 53]. Although the risk of Zn deficiency in populations whose diets consisting primarily of staple crops is well-recognized, the implications of variation in crop Zn concentration for human Zn status have not been documented.

We show that for populations who consume Zn primarily from plant-based foods, an accurate assessment of Zn concentration in local staple crop supplies is necessary in order to predict the prevalence of inadequate dietary Zn intake. Staple crop Zn concentrations reported in the HarvestPlus FCT led to an overestimate of the prevalence of inadequate dietary Zn intake for Ugandan children under the age of 5 who rely largely on home-grown crops, but an underestimate of the prevalence of inadequate dietary Zn intake in children who primarily consume staple crops purchased at market. This discrepancy has widespread implications for our understanding of the global burden of Zn deficiency in young children. While under-estimating the risk of Zn deficiency may lead to inaction and continued loss of life, over-estimating the risk of Zn deficiency may result in misallocation of limited resources [14]. Furthermore, the observed difference between Zn concentration in crops produced on household farms and those sampled at markets suggests greater vulnerability to Zn deficiency in urban areas, as well as in rural areas during the lean season when smallholder households are most dependent on food purchased at market [54, 55, 56]. These patterns of vulnerability are not reflected in the Zn intake estimates based on existing FCTs.

However, more research is needed to understand whether and why staples crops sold in Ugandan markets contain less Zn than staple crops grown on household farms. As noted above, the strength of our analyses is limited by differences between the identity of staple foods sampled from farms (cassava root, maize grain and sorghum grain) and the staples purchased at markets (cassava, maize and millet flour). While we adjust for these differences to the extent possible, variation in food processing methods may still account for some of the differential observed between home-grown and market-purchased staples. It is also possible that agronomic differences contribute to variation in Zn concentrations. A relatively small number of farmers provide the majority of staple crops to sub-Saharan markets [56]. Farmers producing crops for market may use different crop varieties or soil management practices when producing staples for market rather than for household consumption. The Zn content of staples sold at market might also be lower if they have been grown on soil that has been cropped repeatedly with very low inputs to replenish nutrients and organic matter. Our data cannot be used to investigate the underlying cause of different Zn concentrations in staple crops produced on household farms and those sold at markets. However, whether the observed differences are due to processing, inherent crop nutrient density, or other factors, our data suggest that Ugandan children reliant on market-purchased staples are likely to consume less Zn, and FCTs may be misleading if they do not accurately account for lower nutrient density in these foods (Fig 4).

More accurate information regarding the Zn concentration of staple crops in developing countries would likely lead to more accurate estimates of the prevalence of dietary Zn inadequacy. For instance, if high-yielding hybrid cereal varieties are less nutrient-dense and more commonly grown for sale at market than local cereal varieties, this could drive lower Zn intake in urban areas compared to rural areas. Local maps of soil characteristics and soil Zn availability may also be used to assess regional vulnerability to crop Zn deficiency and subsequent dietary inadequacy [57].

Comprehensive, country-specific or sub-national data on crop Zn concentration might also illuminate spatial patterns in crop Zn concentration and human Zn intake. Such data could be used to create country-specific FCTs similar to the one prepared for Ethiopia [30]. Yet in order to determine the extent to which a country- or region-specific FCT would improve our understanding of nutrient intake, it is first necessary to understand the heterogeneity in nutrient content of foods available across the country or region of interest. We make some progress towards this goal in the work presented here, but a much larger sampling effort would be necessary to capture the degree of variation across different foods, locations within the country, and seasonal or annual fluctuations. To capture representative values and better understand this heterogeneity, sampling intensity should be highest for the most variable foods, areas, or times. We believe such an effort would be worthwhile and could improve estimates of micronutrient consumption and shape approaches to nutritional interventions. We hope that our work highlights the need to investigate such variation in food micronutrient concentration, particularly in the case of Zn. Since current estimates of global human Zn status predict that over 2 billion people are affected by Zn deficiency, this work is relevant for a large portion of the world’s population.

### Contributors

LB designed the food frequency survey and data collection procedures, and was the country coordinator for the data collection and household surveys. LB also conducted the statistical analyses, with critical insight and suggestions from RH. Both authors contributed to the writing of the manuscript and approved the final version of the manuscript.

## Supporting information

S1 Fig Portion size picture of mukene. (TIF)

S2 Fig Portion size picture of katogo. (Small Fish) (Cassava and Beans).
(TIF)

S1 Table Food frequency questionnaire. (DOCX)

S2 Table Food and data sources used to estimate dietary zinc intake. * Indicates adjustment to account for nutrient loss due to processing. ** The HarvestPlus FCT lists the same Zn concentration value for millet and sorghum grain.
(TIF)

S1 Data (ZIP)
