# Supplementary figures and images for: Variation in crop zinc concentration influences estimates of dietary Zn inadequacy
Source: PLoS One. 2020 Jul 9;15(7):e0234770. doi: 10.1371/journal.pone.0234770 (PMC7347138; doi:10.1371/journal.pone.0234770)

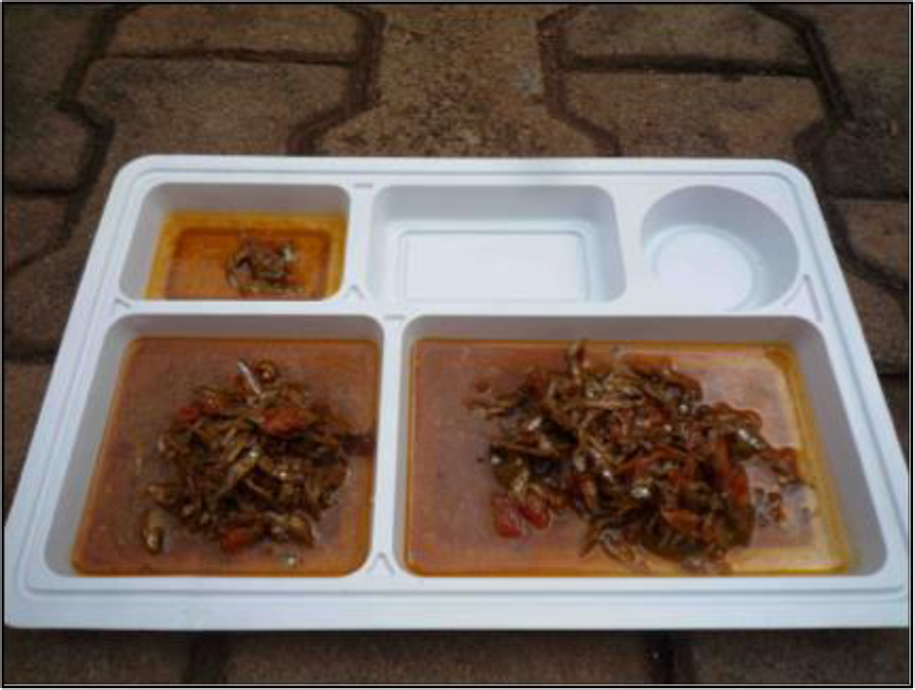

Supplement: S1 Fig — (TIF) [file pone.0234770.s001.tif]

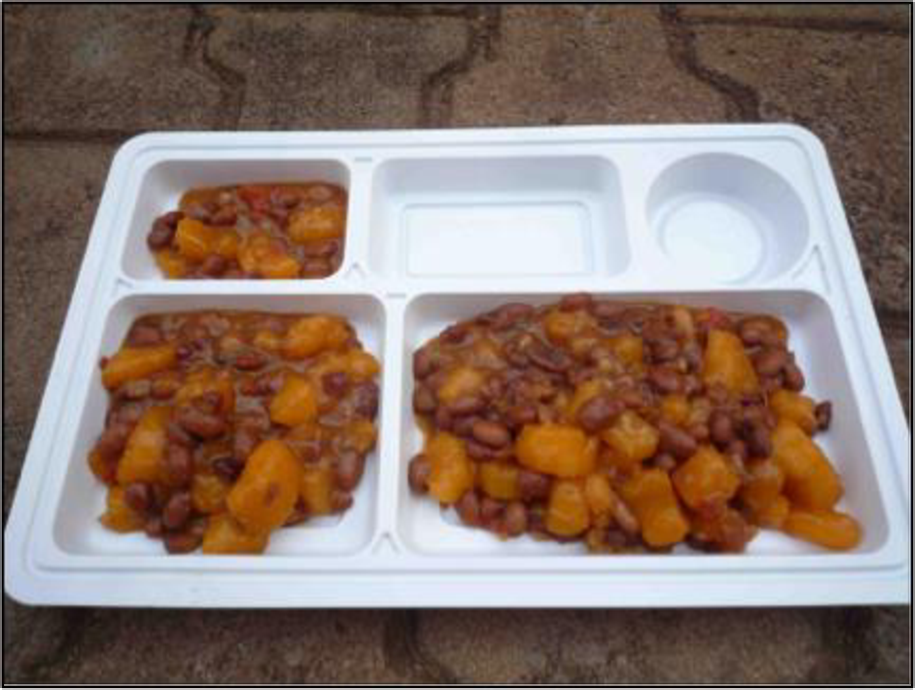

Supplement: S2 Fig — (Small Fish) (Cassava and Beans). (TIF) [file pone.0234770.s002.tif]

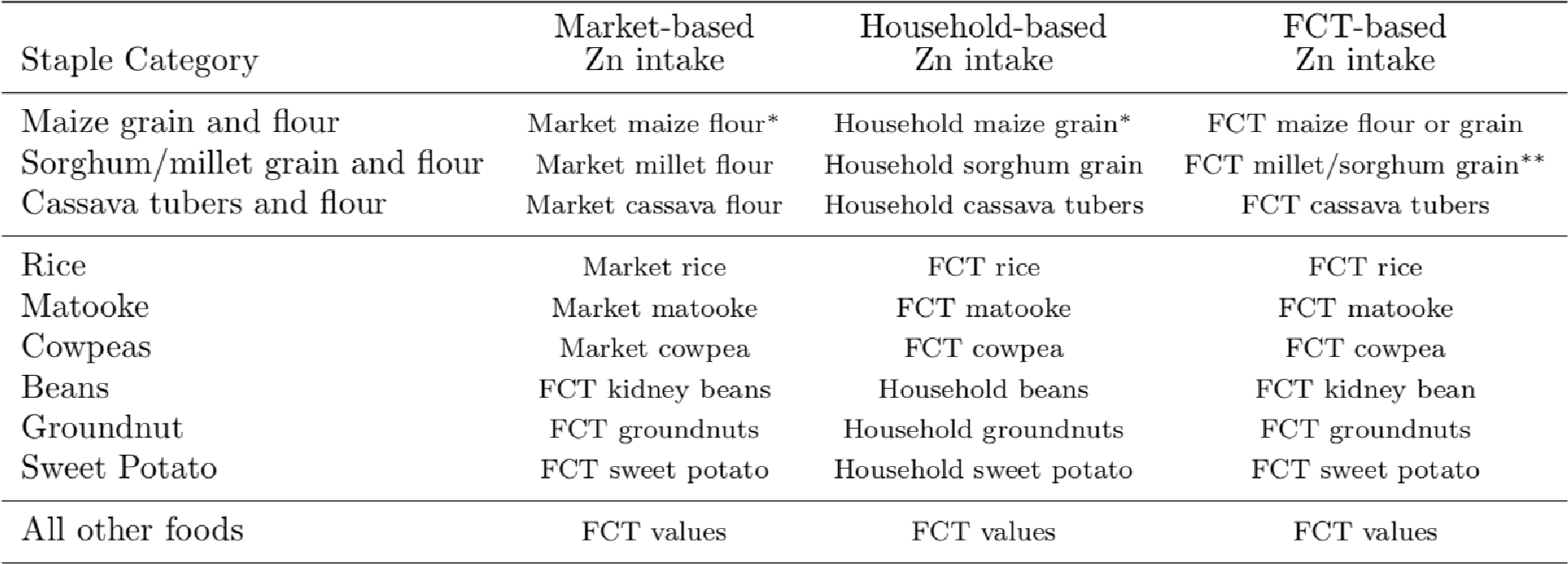

Supplement: S2 Table — * Indicates adjustment to account for nutrient loss due to processing. ** The HarvestPlus FCT lists the same Zn concentration value for millet and sorghum grain. (TIF) [file pone.0234770.s004.tif]
