# Supplementary material for: Variation in crop zinc concentration influences estimates of dietary Zn inadequacy
Source: PLoS One. 2020 Jul 9;15(7):e0234770. doi: 10.1371/journal.pone.0234770 (PMC7347138; doi:10.1371/journal.pone.0234770)
Supplement: S1 Table — (DOCX) [file pone.0234770.s003.docx]

Table A1

|  | | | How many times eaten in the last week? | Average portion size? (small, medium, large) |
| --- | --- | --- | --- | --- |
| Mugoyo/  Amukeke | Sweet potatoes & beans | 1 |  |  |
|  | Sweet potato only | 2 |  |  |
| Katogo | Cassava | 3 |  |  |
|  | Cassava & beans | 4 |  |  |
|  | Cassava & gnuts | 5 |  |  |
|  | Matooke only | 6 |  |  |
|  | Matooke , beans | 7 |  |  |
|  | Matooke, gnuts | 8 |  |  |
| Atap | Posho (maize) | 9 |  |  |
|  | Cassava | 10 |  |  |
|  | Millet | 11 |  |  |
|  | Sorghum | 12 |  |  |
|  | Cassava, millet | 13 |  |  |
|  | Cassava, sorghum | 14 |  |  |
|  | Millet, sorghum | 15 |  |  |
|  | Cassava, millet, sorghum | 16 |  |  |
|  | Sweet potato, millet | 17 |  |  |
|  | Sweet potato, sorghum | 18 |  |  |
|  | Sweet potato, sorghum, millet | 19 |  |  |
| Porridge | Millet | 20 |  |  |
|  | Millet, cow milk | 21 |  |  |
|  | Maize | 22 |  |  |
|  | Maize, cow milk | 23 |  |  |
|  | Maize, soy | 24 |  |  |
|  | Sorghum | 25 |  |  |
|  | Sorghum, cow milk | 26 |  |  |
| Plain  Staples | Cassava | 27 |  |  |
|  | Sweet Potato | 28 |  |  |
|  | Matooke | 29 |  |  |
|  | Maize on cobb | 30 |  |  |
|  | Rice | 31 |  |  |
| Non-Meat  Sauces | Gnut sauce, plain (*north & south*) | 32 |  |  |
|  | Gnut paste w/ veg (*north & south*) | 33 |  |  |
|  | Bean sauce, plain | 34 |  |  |
|  | Cowpea sauce, plain | 35 |  |  |
|  | Peas, fried (*from north*) | 36 |  |  |
|  | Peas, pasted (*from north*) | 37 |  |  |
| Meat Sauces | Fish, boiled/roasted meat | 38 |  |  |
|  | Fish, boiled meat w/ gnut sauce | 39 |  |  |
|  | Mukene, plain | 40 |  |  |
|  | Mukene, w/ gnut sauce | 41 |  |  |
|  | Meat, boiled/roasted meat | 42 |  |  |
|  | Organ meat | 43 |  |  |
| Individual  Food Items | Gnuts, roasted | 44 |  |  |
|  | Mushrooms, plain sauce or w/ veg | 45 |  |  |
|  | Eggs, boiled | 46 |  |  |
|  | Eggs, fried | 47 |  |  |
|  | Milk | 48 |  |  |
|  | Milk Tea | 49 |  |  |
|  | Bread, cakes, mandazi | 50 |  |  |
|  | Avocado | 51 |  |  |
|  | Mangos | 52 |  |  |
